# A systematic review of retinoic acid in the journey of spermatogonium to spermatozoa: From basic to clinical application

**DOI:** 10.12688/f1000research.110510.2

**Published:** 2022-07-27

**Authors:** Ria Margiana, Cennikon Pakpahan, Mulyoto Pangestu

**Affiliations:** 1 Department of Anatomy, Faculty of Medicine, Universitas Indonesia, Jakarta, Indonesia; 2 Master's Programme Biomedical Sciences, Faculty of Medicine, Universitas Indonesia, Jakarta, Indonesia; 3 Andrology Study Program, Faculty of Medicine, Universitas Airlangga, Surabaya, Indonesia; 4 Department of Biomedical Sciences, Faculty of Medicine, Universitas Airlangga, Surabaya, Indonesia; 5 Education Program in Reproduction and Development (EPRD), Department of Obstetrics and Gynaecology, Monash Clinical School, Monash University, Clayton, Australia

**Keywords:** retinoic acid, spermatozoa, male infertility, reproductive health care, clinical application, stem cell

## Abstract

**Background:** Retinoic acid plays an essential role in testicular development and functions, especially spermatogenesis. We have reviewed the role of retinoic acid from basic (molecular) to clinical application.

**Methods:** A search was conducted in the online database including PubMed, Google Scholar, and Scopus for English studies published in the last eight years about this issue. We used the Preferred Reporting Items for Systematic Reviews and Meta-Analyses (PRISMA) guidelines in assessing the studies we are going to investigate.

**Results:** Studies indicated that retinoic acid plays an essential role during pluripotent stem cell migration and lineage commitment, cell differentiation, apoptosis, stem cell number regulation, and maturation arrest in spermatogenic cells. Retinoic acid can also affect related protein expression and signaling pathways at different stages of spermatogenesis. Four studies have applied retinoic acid to humans, all of them in the single-arm observational study. The results look promising but need further research with more controlled study methods, randomization, and large samples.

**Conclusions:** This current systematic review emphasizes a novel retinoic acid mechanism that has not been well described in the literature previously on its functions during the first seven days of spermatogenesis, leading to new directions or explanations of male infertility cause and treatments as a part of reproductive health care.

## Introduction

The process of spermatogenesis is highly complex and involves the production of spermatogonia after the primary male sperm cells undergo meiosis. ^1^ The spermatogonia cells produce spermatocytes which further divide into secondary spermatocytes during meiosis I. The whole process of spermatogenesis takes 72 days in humans and occurs in seminiferous tubules. The process begins with the mitotic division of diploid spermatogonium. ^1^^,^^2^ After primary and secondary spermatocytes divide into spermatids, they form a sperm cell, which is a mature spermatozoon. ^3^ Recent studies have discovered that retinoic acid (RA) facilitates spermatogonia differentiation, suggesting its role in male infertility. ^3^ Therefore, the rationale for this review is to provide the efficacy and the role of RA in infertility among males. Moreover, infertility among the male population is debatable and keeps attracting future studies. Subsequently, infertility treatment falls in the subset of andrology, implying that studies involving the impairment of spermatogenesis resulting from vitamin deficiency, ^3^ among other molecular factors, are of scientific concern. ^3^

To date, there are few reports on the role of RA in male reproduction and spermatogenesis. ^4^ This review discusses several important aspects of the intricate relationship between RA and spermatogenesis, including its synthesis, metabolism, distribution of nuclear receptors and known effects on spermiogenesis during cell differentiation and infertility.

The review has two primary purposes: to assess the available literature on the role of RA on the spermatogenesis process from the spermatogonium to the spermatozoa. As a second point of discussion, this review examines the significance of RA in treating male infertility, emphasizing spermatogenesis.

## Methods

### Information sources and search strategy

We conducted a search on the application of RA in a clinical setting in humans. The researcher searched the most relevant studies on Google Scholar, PubMed, and Scopus. These databases include peer-reviewed journals that focus on molecular and advanced medicine and other health-related topics. The search yielded a sufficient number of results without looking through registrations or reference lists.

The search strategy employed in the review process included keywords, truncation, and Boolean operators. The first approach used keywords that summarized the study’s topic. The keywords used in the search followed the phrase searching approach. For example, “what is the role of RA in spermatogenesis.” The second strategy used for the search comprised the truncation method. The researcher used the truncation (asterisk) plan to save time. In this case, truncation was focused on finding the study’s population. For example, spermat* was used to find spermatogenesis, spermatogonium, spermatozoa. Boolean operators such as “AND, OR, and NOT” were used to narrow, broaden and exclude specific phrases, respectively. For example, the reviewer used a secondary search such as “retinoic acid AND spermatogenesis” to generate accurate studies. We use this keyword to provide wide literature about basic sciences or molecular aspects of RA in spermatogenesis. Meanwhile, to find articles obtaining the clinical use of vitamin A in humans, we used the keywords “retinoic acid” AND “male infertility” AND “experimental study” OR “clinical trial”.

### Eligibility criteria

We collected data using the Preferred Reporting Items for Systematic Reviews and Meta-Analyses (PRISMA) guideline. ^42^ All studies had to meet the following requirements: (1) randomized clinical trial (experimental study), or cohort observational with single-arm study; (2) written in English; (3) examining sperm parameters as output; (4) the study’s full text was available. Studies that did not meet the following criteria were eliminated: (1) the study was not designed in an experimental study or cohort observational; instead, it was designed in the form of a case study; (2) duplicate studies; (3) studies that are not available in full text, main data, or data related to RA administration and sperm parameters; (4) the use of a language other than English; (5) no human subjects were used in the research; (6) articles that are in the form of an abstract or literature review.

### Selection process, and data collection and analysis

We carried out separate electronic searches and retrieved information that matched the terms we were looking for independently. Then we collected together the search results we got into one file. RM and CP conducted critical appraisals one by one on studies that matched our inclusion criteria. We discussed any difficulties that surfaced during the search that produced uncertainty with the other writers. The first author’s name, the year of publication, the study design, the place of origin, the sample size, the sample criteria, the outcomes (sperm parameters, physical examination hormone levels, living birth), and the results were found. All of this information was gathered from each study. We contacted the article’s corresponding author if the required information was absent. We didn’t do quantitative analysis because each study’s statistical analysis was different.

The quality of the studies included in this review was assessed using the Newcastle-Ottawa quality evaluation scale (NOQS). This scale is used to assess the quality of observational studies in order to compile a thorough systematic review. All the included studies were assessed for three crucial components: selection of participants, comparability, and the outcome. The maximum total score for this assessment is nine. In terms of quality, the studies were graded as high (9 stars), medium (7-8 stars), or low (less than 7 stars) available from OHRI.

## Results

**Figure 1.**
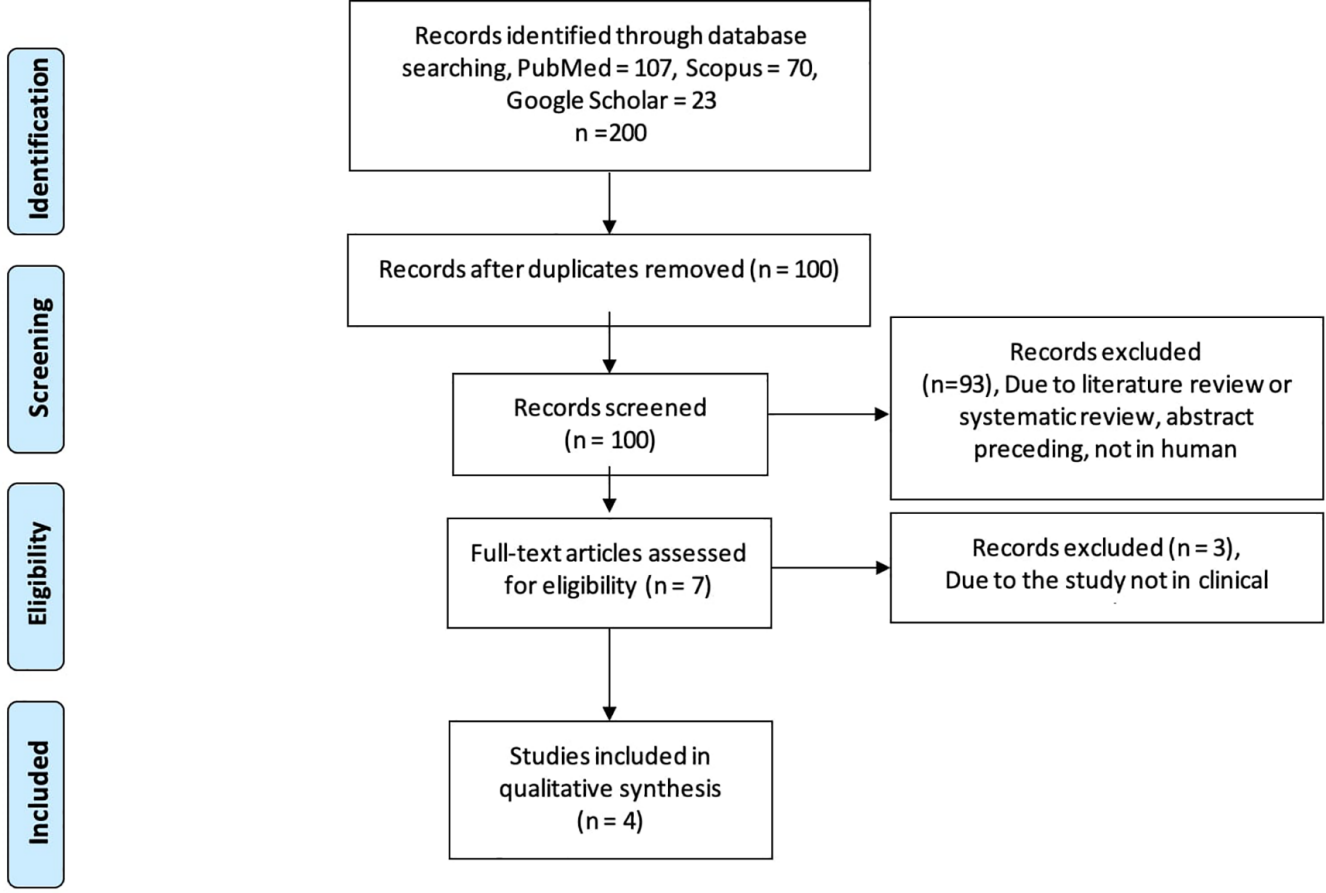
PRISMA flowchart for literature search.

Using the PRSIMA flowchart ^42^ ( Figure 1), we searched the keywords on three databases, including Google Scholar, Scopus, and PubMed databases, we extracted the information about the role of RA in spermatogenesis and the clinical application of RA in humans as a part of infertility treatment. For the clinical application in humans, we found 200 studies relevant to our keywords. Following that, 100 studies were shown to be duplicates. We identified seven potentially relevant papers after screening for English, human research, semen parameters outcomes, and experimental or observational analytic study. Three of the seven studies did not meet our requirements because they do not provide sperm analysis outcomes in clinical settings. Finally, four studies were included in this review ( Table 1). ^5^^–^^8^

**Table 1.** Characteristics and quality studies of clinical application retinoic acid on human.

Author, Year	Study	Country Origin	Sample Size	Sample criteria	Intervention	Outcome	Results	Quality Study *
Amory *et al.*, 2021	Single-arm study	USA	9	Infertile men (21-50 years), azoospermia	Subjects were given self-administered isotretinoin at a dose of 20 mg twice daily for 32 weeks	Analysis semen, pregnancy, and Live birth	From nine patients sperm found in five men who underwent treatment in different weeks. One of these patients achieved a pregnancy and a live birth of a healthy baby.	7
Amory *et al*., 2017	Single-arm study	USA	19	Infertile men (21-50 years), oligo-asthenozoospermia	Subjects received isotretinoin 20 mg by mouth twice daily for 20 weeks.	Analysis semen, physical examinations, pregnancy, and live birth	At the end of treatment, the median (25th, 75th) sperm concentration increased from 2.5 (0.1, 5.9) million/mL at baseline to 3.8 (2.1, 13.0) million/mL (p = 0.006). There were no substantial changes in sperm motility. The morphology of sperm was shown to be improving (p = 0.056). During the trial, there were six pregnancies (three spontaneous and three after intracytoplasmic sperm injection) and five deliveries.	8
Cinar *et al*., 2015	Single-arm study	Turkey	80	Males older than 18 years old, had severe refractory acne, no smoked, consumed alcohol, and genitourinary tract surgery history.	They were given a total dose of 120 mg/kg of systemic isotretinoin over a period of six months.	Analysis semen, hormone levels	The spermiogram parameters all changed in a favourable way (p<0.05). The hormone levels did not alter significantly.	8
Eyada *et al*., 2020	Single-arm study	Egypt	40	Male patients over the age of 18 with normal sperm tests and treated with systemic Accutane for acne vulgaris met the inclusion criteria. Patients who had previously used systemic isotretinoin or who had medical contraindications to it, patients with azoospermia or leukocytospermia, and patients with andrological or medical problems that affected semen parameters were also excluded.	Over the course of 6 months, all patients received a total cumulative dose of 120 mg/kg of systemic isotretinoin.	Analysis semen, sperm ultrastructure assay	It was discovered that there was a statistically significant increase in sperm concentration (million/ml), progressive sperm motility percent, and morphologically normal sperms percent. All sperm ultrastructural metrics and sperm structure dimensions were not significantly modified following isotretinoin use, according to transmission electron microscopy.	8

* Study quality was determined using the Newcastle-Ottawa quality evaluation scale (NOQS) score on the observational study. This scale assesses the study through three aspects, namely selection (recruitment), comparability, and exposure.

The four studies that met our criteria were single-arm observational studies. Two are from the USA, one is from Turkey, and one is from Egypt. The sample group of two studies participating were men who used RA as part of the acne vulgaris treatment. However, there is also one study explicitly conducted on men with azoospermia. The total sample involved in this systematic review is 148 samples. All studies examined semen analysis as an output parameter to assess the success of therapy. However, some consider pregnancy and live birth one of the study outputs. More detailed results from the retrieval study are in Table 1.

## Discussion

The use of RA as a treatment option in male infertility is still limited. The findings from the review show that RA plays a significant role in the spermatogenesis process. Spermatogenesis refers to how spermatogonia develop into mature spermatozoa and constitutes several developmental stages. ^1^ The signal transduction of retinoic acid-binding receptors (RAR) is crucial in this process. This process also involves a series of transcriptional events regulated by various factors such as RAR, which control the initiation of the meiotic process from spermatogonium to primary spermatocytes and control the morphological transformation from round spermatids to elongated spermatozoa. ^1^

Of note, RA is a derivative of vitamin A, and it plays an essential role in spermatogenesis. During spermatogenesis, retinoic acid receptor gamma (RARγ) activates the expression of three genes: luciferase, Crypto, and RAR-related orphan receptor gamma-t (ROR_gamma-t). ^4^ Both luciferase and ROR_gamma-t bind together to activate androgen receptor (AR) functions. AR triggers the expression of 41 genes implicated in spermatogenesis, such as Bos1. Bos1 encodes an enzyme called β-Hydroxysteroid Dehydrogenase Type 1, promoting testosterone synthesis. ^9^ Testosterone is essential for spermatozoa development into mature sperm cells. Furthermore, RA also regulates both hormone and gene expressions. ^10^

RA is essential for the proper development of the adult male’s reproductive system. RA is involved in Sertoli cells differentiation, after which it regulates the later steps of spermatogenesis through the maintenance of germ cell proliferation and differentiation. ^11^ Furthermore, RA promotes the meiotic process by inducing pachytene arrest in the first meiotic prophase. ^12^ The spermatogonium goes on a long journey to mature spermatozoa. During this process, immature germ cells undergo differentiation and transformation by RA in each stage. RA is a derivative of vitamin A, synthesized from chemicals such as retinal that are ingested or formed from other vitamins already present within the body. ^12^

Critically, RA plays an essential role in development, growth and differentiation throughout life. Importantly, for gonadal function and sperm production, in particular, RA is required for spermatogonia to differentiate into spermatozoa. ^13^^,^^14^ Retinoic acid receptor alpha (RARα) and beta (RARβ) are specific receptors found in the testes, both of which have a pivotal role in spermatogenesis. Mutations of RARα have been reported to cause male infertility. ^15^^,^^16^ Scholars evaluated the effect of RA on spermatogenesis and RAR expression in mice models with defective RA signalling. ^15^^,^^16^ They discovered that mice with depleted RA receptors showed a decrease in overall fertility and testicular weight as well as numerous defects such as failure to grow seminiferous tubules, less mature sperm cells, and decreased RAR expression.

**Figure 2.**
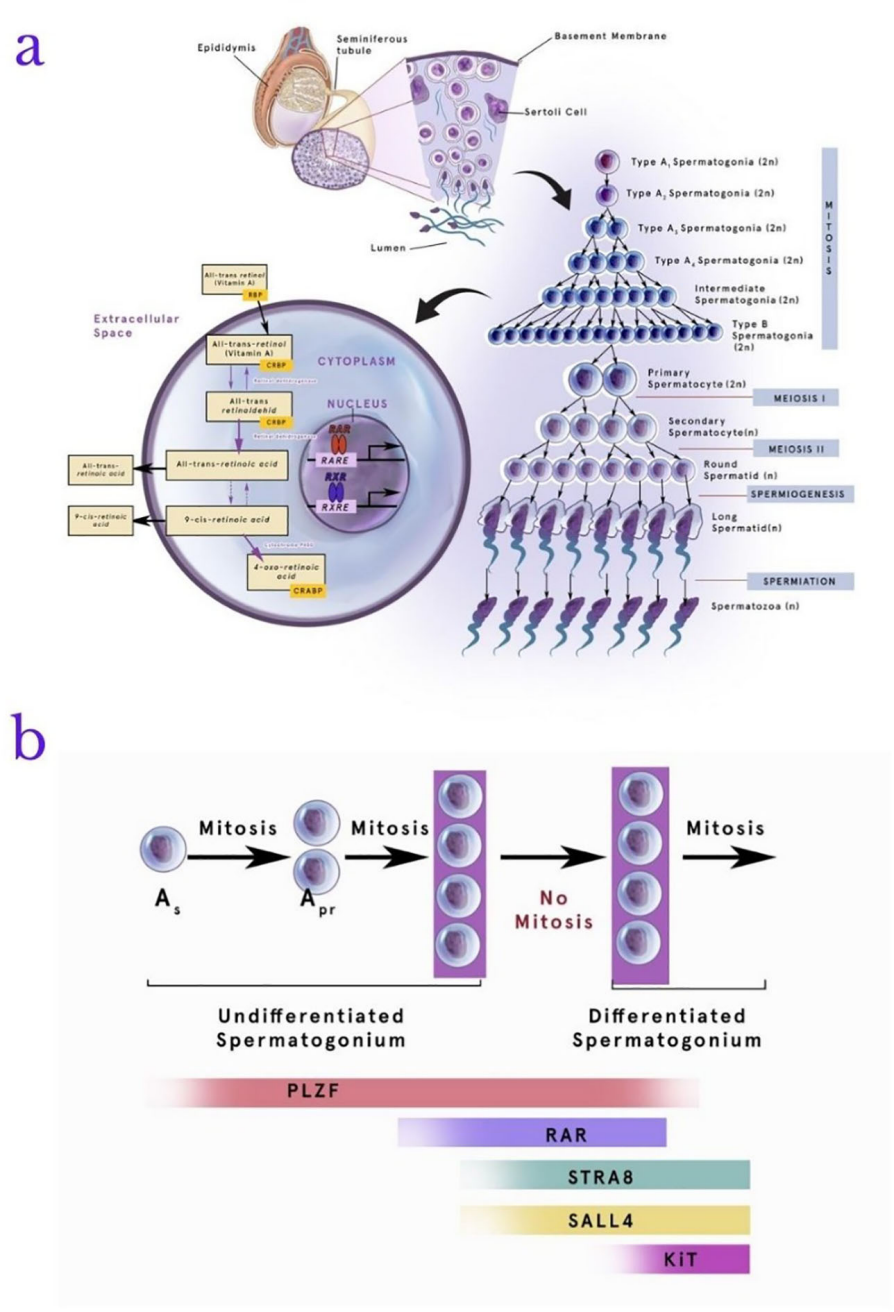
Retinoic acid (RA) metabolism and role in the stages of spermatogenesis. RA plays a role in the process of spermatogenesis. All-trans retinol will enter the cytoplasm and be converted into 4-oxo-retinoic acid as the final product of metabolism. Spermatogonia will utilize this product in the phases of mitosis and meiosis to become spermatozoa (a). In addition, the involvement of RA in spermatogenesis involves several proteins, one of which plays an active role is stimulated by retinoic acid gene 8 (Stra8) together with retinoic acid receptor alpha (RAR-α), Plzf (promyelocytic leukemia zinc finger), Spalt-like 4 (SALL4), and e-KIT. These proteins are dominantly involved in transforming the Apr spermatogonia into intermediate spermatogonia. The absence these proteins interaction with RA may cause the process of mitosis to discontinue (b). RAR-α: retinoic acid receptor alpha, Stra8: stimulated by retinoic acid gene 8, Plzf: promyelocytic leukemia zinc finger, SALL4: Spalt-like 4, RARE: retinoic acid response elements, RXR: retinoid X receptors, RXRE: retinoid X receptors elements.

In the mammalian testis, RA is one of the most important inducers of spermatogenesis. ^17^ In humans, the spermatogenesis process takes 72 days, and RA initiates the undifferentiated spermatogonia. However, it is important to note that RA is produced in the fetal testis, particularly at the mesonephros during the fetal period. RA can prevent meiosis during the fetal period because of CYP26B1 (a metabolizing enzyme of RA) produced by the Sertoli cells. ^17^ Therefore, this implies that RA regulates meiotic divisions in spermatogenesis when in action. It ensures that the spermatogenesis process (I-XII) occurs without interference, resulting in haploid spermatid formation. ^17^ The formed haploid spermatid then becomes spermatozoa, which occurs due to morphological changes in the spermatogenic epithelium. ^17^ Figure 2 below shows the role of RA in the stages of spermatogenesis.

At the start of the cycle, the concentration of RA is low because of the effects of enzymatic inhibitors, especially from stage I-III. The source of RA in this process includes the germ and Sertoli cells. ^17^ The ALDH1A1 and ALDH1A2 catalyse the RA production, increasing concentration from stage VII-VIII. During the VII-VII, a transition occurs from Aa1 to A1 spermatogonia, in which RA induces the process of meiosis. The bidding process occurs at the peak of RA synthesis, where it binds with RAR to activate the downstream expression of genes such as Stra8. ^17^

It has been reported that RA plays a crucial role in promoting meiosis progression, spermatid differentiation and spermiation among male gonad cells. ^18^^–^^22^ In this present systematic review, the retinol-binding protein gene emerged using quantitative real-time polymerase chain reaction (qRT-PCR) analysis and investigated two critical regulatory mechanisms in germ cells: the differentiation and apoptosis of germ cells. ^19^^,^^23^ The results demonstrated that retinol-binding protein (RBP) mRNA expression was upregulated in the RA group compared with the control group after exposure to RA. The systematic review results suggest that RA plays an essential role in regulating testicular function by increasing RBP levels through multiple pathways and processes.

RA is required for the normal growth and development of spermatozoa. Spermatogonium and spermatocyte development require RA signalling, while spermatozoa only use endogenous metabolite concentrations. ^24^^–^^28^ The requirement for retinoids in sperm development, and their ability to restore male fertility when topically administered, highlights the importance of these metabolites during spermatogenesis.

RA is a small hydrophilic signal molecule that plays a role at the earliest stages of spermatogenesis to regulate the expression of factors involved in differentiation and proliferation, like SOX9 or DAZL. ^24^^–^^27^^,^^29^^–^^30^ RA participates in class switching during meiosis and maintains pluripotency and proliferation of stem cells through these essential factors.

RA is required for the initial stages of spermatogenesis. It is required to transition a spermatogonium to a primary spermatocyte and again from a primary spermatocyte to a secondary spermatocyte. ^31^^–^^36^ RA also plays a role in the maturation of spermatozoa, as mutagenesis at specific sites in the RAR prevents the proper maturation of spermatozoa.

RA is required to develop spermatogonia from its precursors to germ cells during fetal development. Once the adult testis has been established, RA becomes redundant for spermatogonia’s continued survival and proliferation. ^32^ In rodents, it was observed that nutritional deprivation could lead to an up-regulation of retinoid synthesis in adult testes. ^32^

Retinoid acid deficiency during prenatal development or after birth can lead to male infertility. It was previously believed that retinoids were no longer needed for male fertility once the adult testis had developed and been established. ^36^ Still, research has shown that when nutrition is low in adult rodents, a retinoid cycle is restored. ^36^^–^^38^ Overall, RA plays an essential role in controlling fertility due to its effects on regulating spermatogenesis through modulating testosterone-dependent gene expression during development via retinoid X receptors.

The use of retinoic acid so far is given orally and topically. Cis-Retinoic Acid is a preparation that is commonly used orally, while tretinoin is topical. ^39^ Peak plasma concentrations have been obtained after 3‐4 hours of use with a half‐life of 1.3‐2.4 hours. ^40^ The topical application usually results in lower plasma concentrations due to poor absorption. ^39^ The primary elimination mechanism was metabolism. The predominant metabolite in plasma was 4‐Oxo‐9‐cis‐retinoic acid, one of four metabolites examined along with all-trans-retinoic acid and 13-cis-retinoic acid, reaching peak plasma levels between 41 and 83 percent of those of 9‐cis‐retinoic acid. ^40^

There are no established reports of cis-retinoid levels in the testes. While the study by Endo et al. in rats reported that RA levels increased twofold (average 20 increase to 50 pmol/g) of the testes after a day of RA injection. ^41^ Still, studies by Amory et al. reported that men who were initially oligoasthenozoospermia and then had their plasma seminal cis retinoid levels checked were undetectable. However, using 13-cis-retinoid as much as 20 mg daily resulted in an average 13-cis retinoic acid concentration of 9.6 ± 2.4 nM in seminal plasma. ^6^ Understanding the great role of RA in the process of spermatogenesis and also the its pharmacokinetics, RA may have a big impact on clinical improvement of male infertility.

Regarding the use of RA in treating male infertility, the four studies that we included conducted trials in men with oligospermia (three studies) and azoospermia (one study). Two studies were conducted on patients with acne vulgaris, ^7^^,^^8^ and it is known that isotretinoin is the treatment of choice for acne. All three studies consistently reported changes in semen parameters in terms of concentration, motility, and morphology. ^7^^,^^8^ Of these three studies, one examined hormone levels and reported no changes. ^7^ Then there was one study that reported whether the effect of using RA gave changes to the ultra-microscopic structure of sperm but found no significant differences. ^8^

In a study using patients with azoospermia, the use of RA was reported by some participants to produce sperm after several weeks of using RA. ^5^ In this study, a surgical sperm retrieval procedure was also carried out. The results found a change in the profile of germ cells in the testes where sperm was collected. This sperm was used in an assisted reproductive technology program with an intracytoplasmic injection procedure, there was one pregnancy, and live birth was successful. ^5^ The results of this study indicate that the use of RA affects the process of spermatogenesis, which may have stopped earlier.

The results of this study support the hypothesis of the role of RA in modulating and initiating the process of spermatogenesis from division to differentiation as we described earlier; however, these studies seem weak in explaining whether these changes occur due to a single role of RA or other factors. Several of these studies have attempted to select samples with biased factors such as smoking, alcohol consumption, and history of genitourinary surgery. However, to get better results in looking at the clinical use of RA in male infertility cases, a randomized clinical control design is the most appropriate. However, this information can be used as a pilot study for further clinical trials, especially to get the most appropriate dose and duration of use.

## Conclusion

RA plays an essential role in activating and modulating the process of spermatogenesis so that mitosis can continue to the final stage of meiosis. The role of RA molecularly in the process of spermatogenesis seems to have a very vital role. However, this argument is still unclear as the use of RA in treatment has not consistently provided a significant effect. Large randomized and blinded studies are needed to prove the benefit of RA in treating male infertility.

## Data availability

All data underlying the results are available as part of the article and no additional source data are required.

### Reporting guidelines

Figshare: PRISMA checklist and flowchart for ‘A Review of Retinoic Acid in The Journey of Spermatogonium to Spermatozoa: From Basic to Clinical Application’ https://doi.org/10.6084/m9.figshare.19404020.v2. ^42^
