# Supplementary material for: A systematic review of retinoic acid in the journey of spermatogonium to spermatozoa: From basic to clinical application
Source: F1000Res. 2022 Jul 27;11:552. Originally published 2022 May 20. [Version 2] doi: 10.12688/f1000research.110510.2 (PMC9345263; doi:10.12688/f1000research.110510.2)
Supplement: Peer review history

# Peer review: Reviewer response for version 2

**DOI:** 10.5256/f1000research.136641.r145610

**Published:** 2022-08-01

**Authors:** Binarwan Halim

**Affiliations:** 1 Faculty of Medicine, Universitas Prima Indonesia, Medan, Indonesia

Based on the revision, I have no further comments to make.

Are the rationale for, and objectives of, the Systematic Review clearly stated?

Yes

Is the statistical analysis and its interpretation appropriate?

Yes

Are sufficient details of the methods and analysis provided to allow replication by others?

Yes

Are the conclusions drawn adequately supported by the results presented in the review?

Yes

Reviewer Expertise:

reproductive endocrinology, andrology, embryology

I confirm that I have read this submission and believe that I have an appropriate level of expertise to confirm that it is of an acceptable scientific standard.

# Peer review: Reviewer response for version 1

**DOI:** 10.5256/f1000research.122128.r142851

**Published:** 2022-07-22

**Authors:** Binarwan Halim

**Affiliations:** 1 Faculty of Medicine, Universitas Prima Indonesia, Medan, Indonesia

Thanks to the authors for trying to make a systematic review of the role of retinoic acid in spermatogenesis. Besides being involved in the role of modulation of gene expression, it can also be applied clinically as the treatment of male infertility. Overall, it was very well written, from the methods through the end of the paper.

 However, I think this study requires minor revision, as it should need to mention the pharmacokinetic and dynamic role of tretionoin administration, as well as its concentration in the testicles.

 After the minor revision, this article should be appropriate for indexing.

Are the rationale for, and objectives of, the Systematic Review clearly stated?

Yes

Is the statistical analysis and its interpretation appropriate?

Yes

Are sufficient details of the methods and analysis provided to allow replication by others?

Yes

Are the conclusions drawn adequately supported by the results presented in the review?

Yes

Reviewer Expertise:

reproductive endocrinology ,andrology,embryology

I confirm that I have read this submission and believe that I have an appropriate level of expertise to confirm that it is of an acceptable scientific standard, however I have significant reservations, as outlined above.

# Response

**Published:** 2022-07-23

**Authors:** Cennikon Pakpahan

**Affiliations:** Airlangga University, Indonesia

Thank you for your suggestions in enriching the information in this paper. We try to revise according to your requests. We think we have explained the dynamic role part in the previous version in the discussion part. While for pharmacokinetics and concentration of retinoic acid in testes, we have added it in the discussion section.

# Peer review: Reviewer response for version 1

**DOI:** 10.5256/f1000research.122128.r141380

**Published:** 2022-07-12

**Authors:** Mathias Abiodun Emokpae

**Affiliations:** 1 Department of Medical Laboratory Science, School of Basic Medical Sciences, University of Benin, Benin City, Nigeria

The study reviewed the roles of RA in modulating and initiating the process of spermatogenesis from primitive cells division, differentiation to matured/functional spermatozoa. RA is involved in the modulation of testosterone dependent gene expression through retinoid receptors and Sertoli cell differentiation. It also reviewed the clinical application of RA in the treatment of male infertility. It also explained the possible mechanisms of action of RA in the process of spermatogenesis. It is a well written paper but requires minor modifications: Keywords should be derived via MeSH on Demand and abbreviations like ALDH1A and CYP may be written in full on first use. The article should be accepted (appropriate) with minor correction.

Are the rationale for, and objectives of, the Systematic Review clearly stated?

Yes

Is the statistical analysis and its interpretation appropriate?

Not applicable

Are sufficient details of the methods and analysis provided to allow replication by others?

Yes

Are the conclusions drawn adequately supported by the results presented in the review?

Yes

Reviewer Expertise:

Reproductive Health studies, human nutrition and sickle cell nephropathy.

I confirm that I have read this submission and believe that I have an appropriate level of expertise to confirm that it is of an acceptable scientific standard.
